# Statin Use in Patients With Cancer: Drug Interaction and Statin Usage

**DOI:** 10.1016/j.jacadv.2025.102259

**Published:** 2025-10-21

**Authors:** Emily Chou, Carlo S. Legasto, Alan K. Chin, Alan H. Baik, Brian C. Schulte

**Affiliations:** a Department of Medicine, University of California Irvine Medical Center, Orange, California, USA; b Department of Pharmaceutical Services, Division of Oncology, University of California, San Francisco, California, USA; c Department of Medicine, Division of Cardiology, Section of Cardio-Oncology, University of California, San Francisco California, USA; d Department of Medicine, Division of Oncology, University of California, San Francisco, California, USA

**Keywords:** cancer survivorship, cardiotoxicity, comorbidities, drug interactions, hyperlipidemia, statin safety

## Abstract

**Background:**

Cardiovascular disease and cancer share common risk factors. Statins, which are widely prescribed for cardiovascular disease prevention, have potential for drug-drug interactions (DDIs) with oncology agents.

**Objectives:**

This study quantifies and characterizes interactions between statins and recently approved oncology agents. Additionally, the study evaluates statin prescribing trends in patients with cancer.

**Methods:**

Food and Drug Administration oncology drug approvals from June 2019 to June 2024 were screened for interactions with 5 commonly prescribed statins using UpToDate Lexidrug. Interaction severity was classified into 5 risk levels. Statin prescribing rates, stratified by cancer type, were characterized through a scoping review of 20 PubMed-indexed observational studies.

**Results:**

Of 138 oncology agents, 33 (23.9%) exhibited DDIs with statins. Simvastatin demonstrated the highest interaction rate (22% [30/138] of oncology drugs), while pravastatin had the fewest (4% [5/138] oncology drugs). Most interactions (88%, 71/81) were classified as level C, requiring monitoring. Contraindicated interactions (level X) were identified for adagrasib, tucatinib, and asciminib. Across the 23,3774 patients in the included studies, statin use was most common in prostate (53%, 57,926/109,140 of patients) and lung cancer (32%, 1403/4344), and least common in liver (8%, 3100/39,722) and breast cancer (10%, 1519/15,078).

**Conclusions:**

Statin-oncology agent DDIs are relatively common but rarely is statin use contraindicated. Frequency of statins use varies according to cancer type. These findings highlight the importance of individualized risk-benefit assessments that consider prognosis, cardiovascular risk, potential drug interactions, and patient preferences to guide statin use in cancer patients.

The relationship between cardiovascular disease (CVD) and cancer has been well characterized.^1^^,^^2^ CVD and cancer have been associated with some common risk factors such as age, obesity, hypertension, diabetes mellitus, and smoking.^2^, ^3^, ^4^, ^5^ Ideal cardiovascular health, as defined by the American Heart Association, is inversely associated with incident cancer.^6^ Exogenous cholesterol has been found to accumulate in cancer tissue, with increased dietary cholesterol associated with incident cancer.^7^, ^8^, ^9^, ^10^ Furthermore, cardiac remodeling may potentiate cancer progression, suggesting early diagnosis and treatment of cardiac disease may improve cancer treatment outcomes.^11^

Statins are commonly prescribed for primary and secondary prevention of CVD and major adverse cardiovascular events. The time to benefit for primary prevention of major adverse cardiovascular events per 100 adults aged 50 to 75 years old is over 2 years.^12^ Previous studies have assessed the risks and benefits of discontinuing statin therapy in the setting of life-limiting disease.^13^^,^^14^ A study in patients with life expectancy of less than 1 year and Karnofsky Performance Status of <80% found that patients who discontinued statin therapy had better quality of life and saved $716.46 per patient over 212.6 days.^13^ Nonetheless, statins are often prescribed and filled by patients with life-limiting illnesses.^15^^,^^16^ The recognition of a life-limiting illness does not necessarily influence reduction in statin use in the last 6 months of life^15^ and one study reported that 31% of patients with cancer filled a statin prescription within 30 days of death.^16^

In contrast, several cancer-directed treatments, such as androgen deprivation therapies, antiestrogen therapies, anthracyclines, and tyrosine kinase inhibitors, are associated with hyperlipidemia.^1^ Therefore, patients with longer life-expectancies would likely benefit from continued lipid monitoring and appropriate statin therapy to reduce cholesterol levels and mitigate CVD and cancer progression.

However, drug-drug interactions (DDIs) are a significant concern in oncology. Polypharmacy is prevalent among cancer patients, who commonly receive complex medication regimens including systemic cancer-directed therapy, supportive therapies, and medications for chronic conditions. For example, a study of Southwest Oncology Group clinical trials found that 28.7% of subjects had DDIs classified as moderate or higher.^17^ Some oncologic agents interact with critical pathways in statin metabolism (for example through CYP3A4 metabolism); however, the extent and distribution of these interactions remain incompletely characterized. Even modest reductions in first-pass statin metabolism or CYP3A4 inhibition can greatly increase systemic statin levels, elevating the risk of myopathy and life-threatening rhabdomyolysis.^18^ Conversely, interactions with statins may directly and indirectly impact efficacy and toxicity profiles of cancer-directed agents.^19^ These considerations are crucial given the narrow therapeutic indices of many oncologic therapies and the increased risk of adverse drug effects.^19^

Therefore, this study aims to quantify and characterize interactions between statins and Food and Drug Administration (FDA)-approved oncologic agents approved between 2019 and 2024 and to examine frequency of use of statin in cancer patients.

## Methods

### Statin and oncology drug interactions

FDA oncology drug approvals from June 27, 2019, to June 27, 2024, were indexed from the FDA's website on oncology and hematologic malignancies approval notifications.^20^ This time frame was selected at the time of project initiation to represent recently approved oncologic agents. Two hundred seven approvals for 138 unique oncology agents were identified. DDIs between oncology agents and 5 most prescribed statins—rosuvastatin, atorvastatin, simvastatin, pravastatin, and lovastatin—were evaluated using UpToDate Lexidrug,^21^^,^^22^ a widely used and comprehensive tool for identifying DDIs. DDI severities were recorded according to the risk ratings designated by Lexidrug (Table 1).^23^

**Table 1:** Lexidrug Online Interaction Risk Rating Categories and Descriptions

Risk Rating	Action	Description
A	No interaction	Data have not demonstrated either pharmacodynamic or pharmacokinetic interactions between the specified agents
B	No action needed	Data demonstrate that the specified agents may interact with each other, but there is little to no evidence of clinical concern resulting from their concomitant use.
C	Monitor therapy	Data demonstrate that the specified agents may interact with each other in a clinically significant manner. The benefits of concomitant use of these 2 medications usually outweigh the risks. An appropriate monitoring plan should be implemented to identify potential negative effects. Dosage adjustments of one or both agents may be needed in a minority of patients.
D	Modify regimen	Data demonstrate that the 2 medications may interact with each other in a clinically significant manner. A patient-specific assessment must be conducted to determine whether the benefits of concomitant therapy outweigh the risks. Specific actions must be taken to realize the benefits and/or minimize the toxicity resulting from concomitant use of the agents. These actions may include aggressive monitoring, empiric dosage changes, choosing alternative agents.
X	Avoid combination	Data demonstrate that the specified agents may interact with each other in a clinically significant manner. The risks associated with concomitant use of these agents usually outweigh the benefits. These agents are generally considered contraindicated.

Lexicomp. Lexi-Interact Data Fields. June 27, 2024. http://webstore.lexi.com/Information/Product-Information/Lexi-Interact-Fields#:∼:text=Severity%20indicators%20include:%20Minor%20(effects,may%20be%20included%20as%20well.

### Scoping review of statin prescribing patterns across cancer types

**Figure 1:**
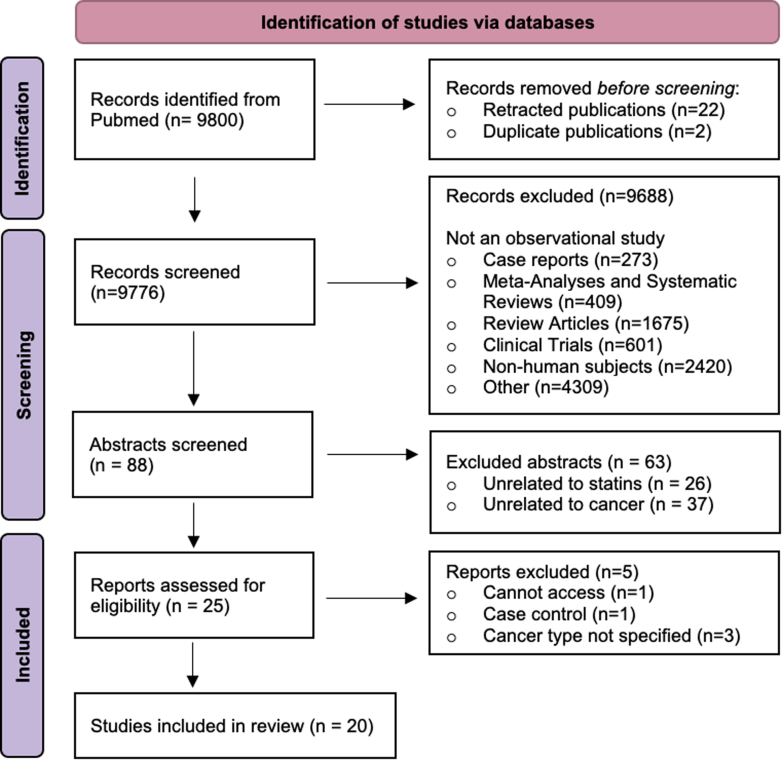
Preferred Reporting Items for Systematic Review and Meta-Analysis Extension for Scoping Reviews Flow Diagram The process of identifying, screening, and selecting studies for inclusion in the scoping review.

A scoping review was performed to characterize statin prescribing rates across patients diagnosed with different cancer types. This scoping review is reported in accordance with the Preferred Reporting Items for Systematic Review and Meta-Analysis Statement Extension for Scoping Reviews guidelines (Figure 1).^24^ Screening and data extraction were conducted. While predefined inclusion and exclusion criteria were used to minimize subjectivity, the absence of duplicate independent review may introduce selection and extraction bias. A single database search was considered adequate given the descriptive nature of this scoping review. PubMed was searched in September 2024 with the following criteria: “(statin OR rosuvastatin OR atorvastatin OR pravastatin OR fluvastatin OR lovastatin OR simvastatin OR HMG-CoA reductase inhibitor) AND (adenocarcinoma OR cancer OR carcinoma OR oncology OR sarcoma OR leukemia OR lymphoma OR malignancy OR tumor OR melanoma OR myeloma OR neoplasm OR neuroblastoma OR dysplasia).” Exclusion criteria included systematic reviews, clinical trials, review articles, case reports, nonhuman subjects, and unrelated articles. Inclusion criteria included observational studies pertaining to statin use in patients with cancer. The review was limited to English-language articles with no restriction on publication year, up to September 2024.

Studies meeting inclusion criteria were included in syntheses based on availability of relevant data. The extracted data included the type of cancer, number of patients prescribed statins, and total number of patients with the specific cancer diagnosis. Additional variables including age, cancer stage, statin type, treatment duration, DDIs, and statin indication were sought post hoc but were inconsistently reported. Data preparation involved aggregating patient counts by cancer type to calculate the proportion of statin users within each category; no additional data transformation was performed. Because no means or variance measures were reported, statistical pooling and meta-analysis were not performed, making SDs not applicable and thus not reported. Included studies were assessed for quality using the Newcastle-Ottawa Scale (Supplemental Table 2).^25^ Given the descriptive nature of this scoping review without meta-analysis, no formal assessment of reporting bias or certainty of evidence was performed. This scoping review was not prospectively registered, and no protocol was prepared prior to conducting the review.

### Statistical analysis

All analyses were performed using Microsoft Excel. Summary data are presented as percentages. Chi-square tests were used to assess differences in in the frequency of oncology drug-statin interactions stratified by statin type, compare rates of statin use among different cancer types, and compare rates of statin use across cancer stages. Statistical significance was defined as a 2-sided *P* value <0.05.

### Ethical approval

This study did not involve the use of human or animal subjects and therefore did not require review or approval by an Institutional Review Board or ethics committee.

## Results

### Statin and oncology agent interactions

**Figure 2:**
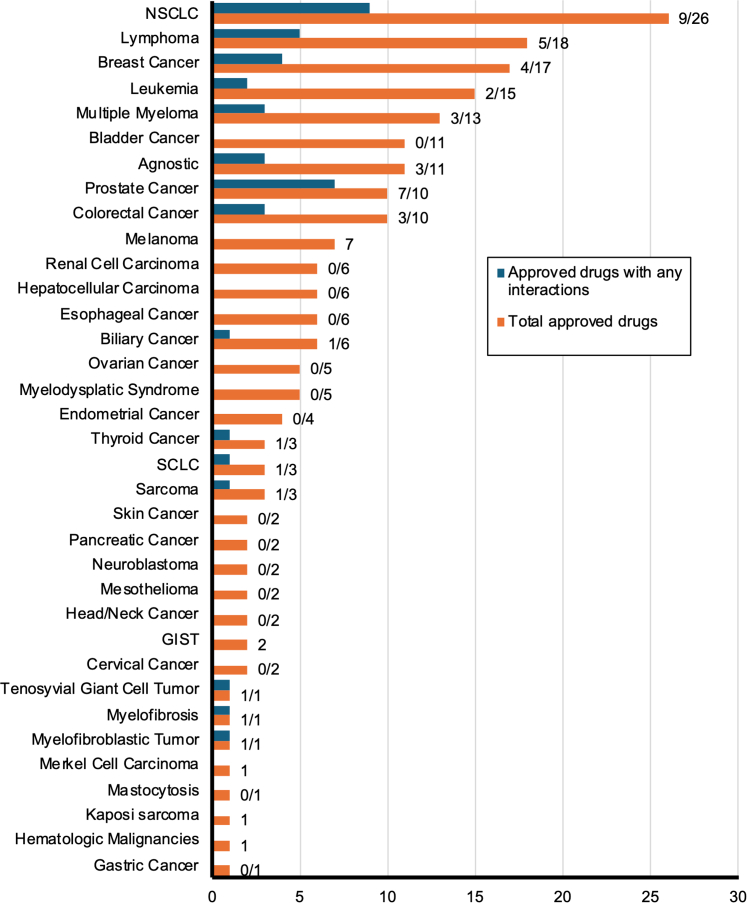
FDA-Approved Oncology Drugs per Indication and Interactions With Commonly Prescribed Statins 207 FDA approvals for oncology drugs from June 27, 2019, to June 27, 2024, were recorded and categorized by their specific indications. DDIs between each oncology agent and the top 5 most prescribed statins—rosuvastatin, atorvastatin, simvastatin, pravastatin, and lovastatin—were evaluated using UpToDate Lexi-Drug. Labels indicate the number of approved drugs with any level of interaction with a statin vs the total number of approved drugs for each indication. DDIs = drug-drug interactions; FDA = Food and Drug Administration; GIST = gastrointestinal stromal tumor; NSCLC = non–small cell lung cancer; SCLC = small cell lung cancer.

Out of 138 approved oncology agents, 33 were found to have any degree of interaction with rosuvastatin, atorvastatin, simvastatin, pravastatin, and lovastatin (Figure 2). Agents approved for the treatment of non–small cell lung cancer (NSCLC) had the highest number of interactions at 9 out of 26 agents. Agents approved for the treatment of prostatic adenocarcinoma had the highest proportion of interactions, at 7 out of 10 agents. Other indications with notable interactions included lymphoma (5 out of 18), breast cancer (4 out of 17), leukemia (2 out of 15), multiple myeloma (3 out of 13), agnostic indications (3 out of 11), and colorectal cancer (CRC) (3 out of 10).

**Figure 3:**
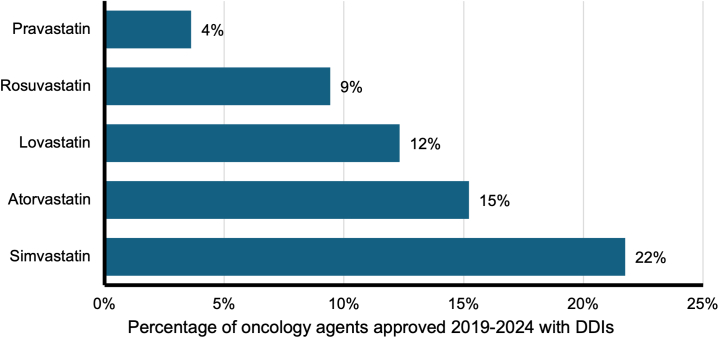
Interactions Between Statins and Recently Approved Oncology drugs Interactions between the 5 most commonly prescribed statins and oncology drugs approved from 2019 to 2024 were quantified. Abbreviation as in Figure 2.

Interactions with specific statins were quantified as well (Figure 3). There was a statistically significant difference in interaction rates among the 5 statins (*P* < 0.001). Simvastatin had the highest interaction rate, affecting 30 of 138 recently approved oncology agents (22%), while pravastatin had the fewest interactions, with 5 of 138 agents (4%).

**Figure 4:**
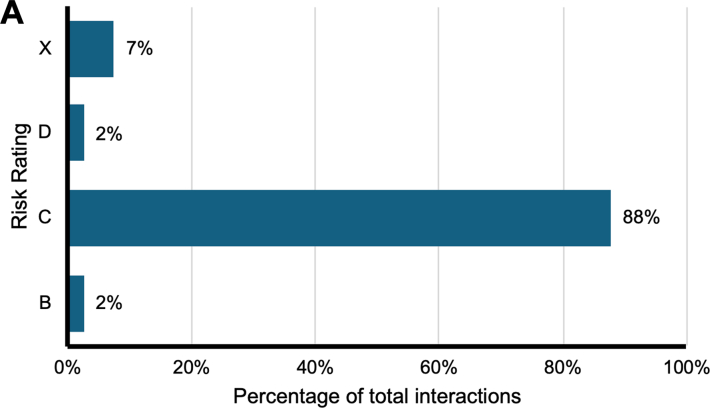

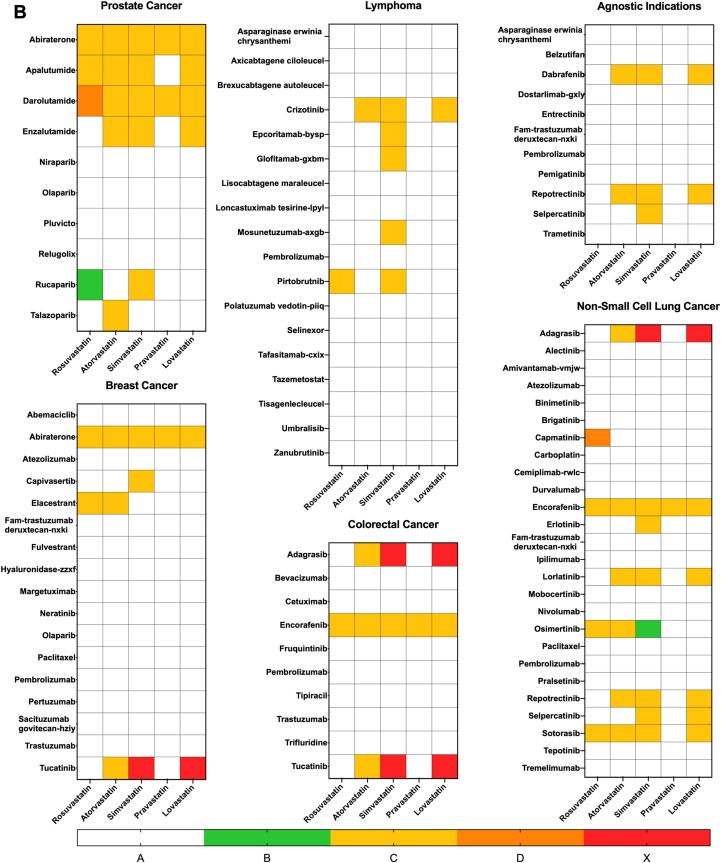
Risk Ratings of Interactions Between Statins and Approved Oncology Drugs (A) Distribution of risk ratings for all interactions between statins and approved oncology drugs. (B) 6 indications were identified to have approved agents with the most interactions with statin drugs. Risk ratings were recorded based on Lexidrug categories as described in Table 1.

The nature of interactions was characterized using risk ratings designated by Lexidrug. Only pharmacokinetic or pharmacodynamic DDIs were evaluated. Of 81 interactions between statins and oncology drugs, 71 (87%) were level C interactions, 6 (7%) were level X, 2 (2%) were level B, and 2 (2%) were level D (Figure 4A). Although drugs approved for prostatic adenocarcinoma had the highest rates of interactions, there was 1 level B, 18 level C, and 1 level D interactions (Figure 4B). Drugs approved for lymphoma exhibited 8 level C interactions and drugs approved for agnostic indications had 7 level C interactions. Agents approved for NSCLC had 1 level B, 21 level C, 1 level D, and 2 level X interactions. Similarly, breast cancer agents exhibited 9 level C interactions and 2 level X interactions. CRC agents had 7 level C interactions and 4 level X interactions (Figure 4B).

Level X interactions involved adagrasib approved for NSCLC and CRC, tucatinib for breast cancer and CRC, and asciminib for chronic myeloid leukemia (CML). Notably, only simvastatin and lovastatin interacted with the included oncology agents at level X severity, apart from asciminib which also interacts with rosuvastatin and atorvastatin with level X severity (Supplemental Table 1).

### Rates of statin use in patients with cancer

**Figure 5:**
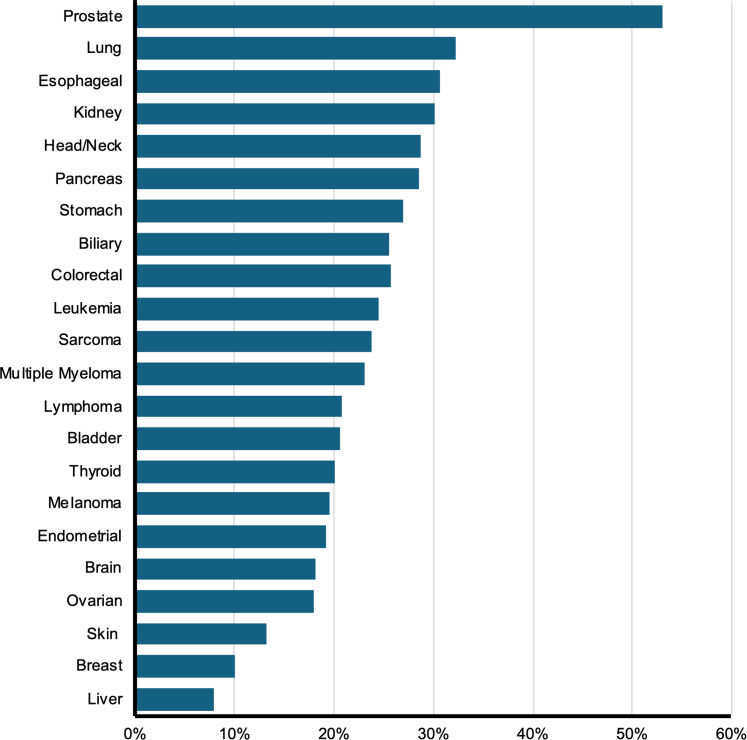
Rates of Statin Use in Cancer Patients by Cancer Types A scoping review was performed to identify observational studies with available data on statin use rates in cancer patients.

A scoping review was performed to assess rates of statin use based on cancer type. Initial PubMed search identified 9800 studies, of which 24 were removed as duplicates or retracted studies. Eighty-eight abstracts were identified as observational studies and were eligible for title and abstract review. Twenty-five were selected for full-text review; 5 were excluded, leaving 20 studies included in this analysis (Figure 1).^26^, ^27^, ^28^, ^29^, ^30^, ^31^, ^32^, ^33^, ^34^, ^35^, ^36^, ^37^, ^38^, ^39^, ^40^, ^41^, ^42^, ^43^, ^44^, ^45^ Study details are delineated in Table 2. Additional characteristics are reported in Supplemental Tables 2 to 4. The studies were drawn from diverse sources, including national health databases, cancer registries, observational cohorts, and single institutions across several countries. Across the 20 included studies, prostate cancer was the most commonly reported cancer type (10 studies), followed by colorectal (9), lung (6), and breast (5). Conversely, certain cancers such as thyroid, sarcoma, biliary, and endometrial cancers were underrepresented. Of the 22 cancer types included, rates of statin use differed significantly across cancer types (*P* < 0.001). Fifteen cancer diagnoses were associated with rates of statin use of 20% or higher. Statin use was highest in patients with prostate and lung cancer (53% and 32%, respectively) (Figure 5). These were followed by esophageal and kidney cancer (31% and 30%). Patients with hepatocellular carcinoma used statins at the lowest rate. Four studies reported specific statins prescribed (Supplemental Table 4). Atorvastatin (619 of 1855 total patients), simvastatin (568), and pravastatin (272) were the most frequently used. Lovastatin (195), fluvastatin (119), rosuvastatin (28), and pitavastatin (9) were less commonly reported.

**Table 2:** Characteristics of Observational Studies With Rates of Statin Use in Cancer Patients

First Author, Year	Data Source	Cancer Type	Statin Users	Statin Nonusers	% Statin Users
Lötsch et al,^25^ 2014	Vienna Cancer and Thrombosis Study at the Medical University of Vienna, Austria, 2003-2011	Brain	20	162	11.0%
		Breast	18	181	9.0%
		Colorectal	14	132	9.6%
		Gastric	6	51	10.5%
		Kidney	7	33	17.5%
		Lung	38	177	17.7%
		Lymphoma	17	220	7.2%
		Multiple myeloma	3	35	7.9%
		Pancreas	8	77	9.4%
		Prostate	27	121	18.2%
Chan et al,^26^ 2015	Prostate cancer patients from the Health Professionals Follow-Up Study, 1992-2008	Prostate	685	3,264	17.3%
Shao et al,^27^ 2015	National Health Insurance claims database and cancer registry databases of The Collaboration Center of Health Information Application, Taiwan. Hepatocellular carcinoma patients treated 2001-2010 and followed to 2012	Liver	1988	18,212	9.8%
Chen et al,^28^ 2016	Brain cancer patients from the National Health Insurance Bureau in Taiwan, 2004-2011	Brain	32	213	13.1%
Wang et al,^29^ 2016	Women’s Health Initiative Observational Study, recruited 1993-1998	Nonmelanoma skin cancer	1,529	10,026	13.2%
Wu et al,^30^ 2016	Taiwan National Health Insurance Research Data linked to the Taiwan Cancer Registry, 2001-2012	Liver	934	17,958	4.9%
Mikkelsen et al,^31^ 2017	Prostate cancer patients at 2 Danish Urological Departments, 2007-2013	Prostate	141	396	26.3%
Palumbo et al,^32^ 2017	Prostate cancer patients at the University of Perugia, 2009-2014	Prostate	55	195	22.0%
Emilsson et al,^33^ 2018	Surveillance, Epidemiology, and End Results (SEER)-Medicare database (2007-2009)	Bladder	49	819	5.6%
		Breast	289	5,547	5.0%
		Colorectal	192	3,491	5.2%
		Prostate	367	5,471	6.3%
Anderson-Carter et al,^34^ 2019	Prostate Cancer patients in the national Veterans Affairs database, 2000-2008 and followed to 2016	Prostate	53,360	33,986	61.1%
Fransgaard et al,^35^ 2019	National Clinical Registry of the Danish Colorectal Cancer Group, 2003-2015	Colorectal	386	1861	17.2%
Jiménez-Vacas et al,^36^ 2020	Prostate cancer patients from the Reina Sofia University Hospital	Prostate	30	45	40.0%
Majidi et al,^37^ 2020	Ovarian Cancer Prognosis and Lifestyle study, Australian women diagnosed with ovarian cancer from 2012-2015 and followed for 5-8 years	Ovarian	200	755	20.9%
Fernandez et al,^38^ 2021	Head and neck squamous cell carcinoma patients from Walter Reed National Military Medical Center, the University of Rochester Medical Center, and an observational clinical study conducted by the National Institutes of Health in partnership with Johns Hopkins University	Head and neck	113	164	40.8%
Okada et al,^39^ 2021	Follow-up observational study of the Japan Primary Prevention of Atherosclerosis with Aspirin for Diabetes trial	Breast	3	14	17.6%
		Colorectal	14	44	24.1%
		Gastric	9	37	19.6%
		Liver	1	23	4.2%
		Lung	2	3	40.0%
		Pancreas	6	16	27.3%
		Prostate	4	16	20.0%
Rossi et al,^40^ 2021	Patients with metastatic non–small cell lung cancer at S. Andrea Hospital Sapienza Rome University, 2015-2020	Lung	88	74	54.3%
Chung et al,^41^ 2022	Taiwan National Health Insurance Research Database, 2001-2008	Colorectal	362	956	27.5%
Pourlotfi et al, 2022	Swedish Colorectal Cancer Register, 2007-2016	Colorectal	5,896	13,222	30.84%
Okamoto et al,^43^ 2023	Colorectal cancer patients at the University of Tokyo Hospital	Colorectal	55	195	22.0%
Lin et al,^44^ 2024	United Kingdom Biobank, recruited 2006-2010 and followed up to 2021	Biliary	75	218	25.6%
		Bladder	370	791	31.9%
		Brain	167	612	21.4%
		Breast	1,209	7,817	13.4%
		Colorectal	1,423	4,275	25.0%
		Endometrial	293	1,234	19.2%
		Esophageal	308	695	30.7%
		Gastric	198	492	28.7%
		Head and neck	245	722	25.3%
		Kidney	467	1,069	30.4%
		Leukemia	318	981	24.5%
		Liver	177	429	29.2%
		Lung	1,275	2,687	32.2%
		Lymphoma	522	1828	22.2%
		Melanoma	555	2,294	19.5%
		Multiple myeloma	200	645	23.7%
		Ovarian	145	821	15.0%
		Pancreas	375	878	29.9%
		Prostate	3,257	7,720	29.7%
		Sarcoma	98	314	23.8%
		Thyroid	87	348	20.0%

Okada et al explicitly assessed primary prevention, while others did not differentiate between primary and secondary atherosclerotic cardiovascular disease (ASCVD) prevention. Duration of statin use was also largely not specified. Chan et al reported 214 patients with prostatic adenocarcinoma used statins for 1 to <6 years and 217 for >6 years, while Wang et al reported 417 patients with skin cancer, used statins for <1 year, 558 for 1 to <3 years, 291 for 3 to <5 years, and 263 for >5 years. DDIs were not reported in any of the included studies.

## Discussion

### Managing statin-oncology drug interactions

We demonstrate the rate of interactions between statins and cancer-directed agents approved between 2019 and 2024 is 23.9% (33 of 138) (Figure 2). These interactions are not homogenous across statin types. Pravastatin exhibited the lowest interaction rates, while simvastatin demonstrated the highest rate of interaction (Figure 3). Statin use was prominent in prostate and lung cancer patients and least in liver and breast cancer patients (Figure 5). These findings underscore the importance of individualizing statin therapy based on prognosis, cardiovascular risk, and DDIs.

Contraindicated combinations between statins and oncology drugs are rare, with 7% of interactions identified as contraindicated combinations (Figure 4A). Eighty-eight percent of statin-oncology DDIs were assigned risk ratings of C, meaning the benefits of concomitant therapy outweigh the risks and patients should be monitored for clinically significant interactions (Figure 4A).

In instances where statin efficacy may be compromised due to interactions, switching to an alternative agent or attempting dose reduction is reasonable rather than discontinuing therapy altogether. Lorlatinib was approved for the treatment of anaplastic lymphoma kinase- or c-ros oncogene 1-positive NSCLC based on the *CROWN Trial* (NCT03052608), but 72% of patients developed hypercholesterolemia with the majority necessitating statin treatment.^46^ Lorlatinib, a moderate cytochrome P450 3A4 (CYP3A4) inducer, may ameliorate the efficacy of statins metabolized by CYP3A4 pathways. Conversely, adagrasib and tucatinib, strong CYP3A4 inhibitors, may elevate serum concentrations of statins, increasing risk of serious adverse events.^47^ In these cases, an alternative statin can be used that avoids CYP3A4 metabolism such as fluvastatin, rosuvastatin, pitavastatin, or pravastatin. Nonstatin lipid-lowering medications such as ezetimibe and proprotein convertase subtilisin/kexin type 9 inhibitors can be considered, as ezetimibe is metabolized through glucuronidation and proprotein convertase subtilisin/kexin type 9 inhibitors are eliminated through lysosomal degradation, thus having a lower likelihood for DDIs.^48^^,^^49^

Though not validated in patients with cancer specifically, the 10-year ASCVD risk calculator could be used to assist decision-making on statin use for primary prevention.^50^ Statins carry a risk of rhabdomyolysis, which is also associated with multiple anticancer agents, necessitating cautious statin selection and monitoring.^51^^,^^52^ Conversely, several anticancer drugs, including anthracyclines and immunotherapies, are associated with cardiotoxicity, where statin therapy may offer protective benefits.^53^, ^54^, ^55^

For patients with CVD risk factors and malignancies with an indolent course, statin continuation may be of significant benefit. Survival in patients with CML is particularly lengthy with tyrosine kinase inhibitors such as imatinib. Long-term treatment with imatinib resulted in a 10-year median overall survival (mOS) of 83.3%.^56^ Patients receiving treatment for CML are expected to lose <3 life-years.^57^ Asciminib, approved for Philadelphia chromosome-positive CML, is contraindicated in combination with rosuvastatin and atorvastatin and demonstrates level C interaction with other statins (Supplemental Table 1).^58^ However, considering the life expectancy of CML patients, alternate statins should be continued based on estimated CVD risk.^1^

### Rates of statin use across cancer types

Patients with prostate cancer exhibit the highest rate of statin use at 53%. This is likely in part due to overlapping prevalence of prostate cancer and dyslipidemia in older males.^59^ Furthermore, androgen deprivation therapies may elevate low density lipoprotein and triglyceride levels, increase visceral and subcutaneous fat, and promote insulin resistance, contributing to heightened ASCVD risk.^60^, ^61^, ^62^ Current management of these adverse effects includes high-intensity statin therapy and aggressive management of CVD risk factors.^63^

Agents prescribed for prostate cancer demonstrate the highest rates of interaction with statins (70%) (Figure 2). However, all interactions were of grade C or lower, barring the grade D interaction between darolutamide and rosuvastatin. Darolutamide is approved for castration-resistant prostatic adenocarcinoma, with mOS exceeding 3 years.^59^^,^^64^ Given the mOS and risk of CVD in this population, the benefits of continuing statin therapy frequently exceed the risks of potential DDIs, particularly in those with preexisting CVD.

Statin use is also prevalent in lung cancer patients, at 32%. Smoking increases the risk of both lung cancer and CVD mortality, and in one longitudinal study, patients with lung cancer were found to have 2 to 4 times increased risk of CVD.^65^^,^^66^ In contrast, patients with hepatocellular carcinoma exhibited the lowest rate of statin use at 8%, possibly attributed to concerns of hepatotoxicity with statins, and the relatively poor prognosis compared to other malignancies.^67^

### Limitations and future directions

Despite the valuable insights provided by this study, several limitations should be acknowledged. The scoping review was conducted solely using PubMed, which may have limited the breadth of literature retrieved compared to a multidatabase search. DDIs were assessed using only UpToDate Lexi-Drug, which may not capture all potential interactions or emerging evidence from other sources. Additionally, this review was not prospectively registered, reflecting its exploratory scope. The review was conducted by a single reviewer, which may introduce bias in study selection and data extraction. This study is based on observational data, which may introduce selection bias and potentially overestimate statin use among cancer patients. The focus on FDA-approved oncology drugs from the past 5 years was intended to capture current therapies but may have excluded older treatments with relevant interactions. Finally, some findings are derived from pooled data with relatively small sample sizes, which may affect the precision and generalizability of the results. Only 1 study explicitly assessed statin use for primary prevention of ASCVD; the remaining studies did not distinguish between use for primary and secondary prevention.^40^ Five papers reported statin use stratified by cancer stage (Supplemental Table 3). Additionally, heterogeneous age reporting across studies limits comparison of age effects on statin use (Supplemental Table 1). These limitations highlight the need for cautious interpretation and further research.

## Conclusions

In this study, we demonstrate that statin-oncology agent DDIs are relatively common; however, statin use is rarely contraindicated. Frequency of statin use differs in patients with various types of cancers. These findings highlight the importance of individualized risk-benefit assessments when deciding upon statin use in cancer patients (Central Illustration).

Perspectives **COMPETENCY IN MEDICAL KNOWLEDGE:** In patients with cancer on statin medications, only a small subset of DDIs are considered strict contraindications and most can be managed with alternative statin selection or dose modifications.

**TRANSLATIONAL OUTLOOK:** Further research is needed to better understand the indications of statin therapy in patients with cancer and the absolute reduction in cardiovascular events and disease progression.

**Central Illustration:**
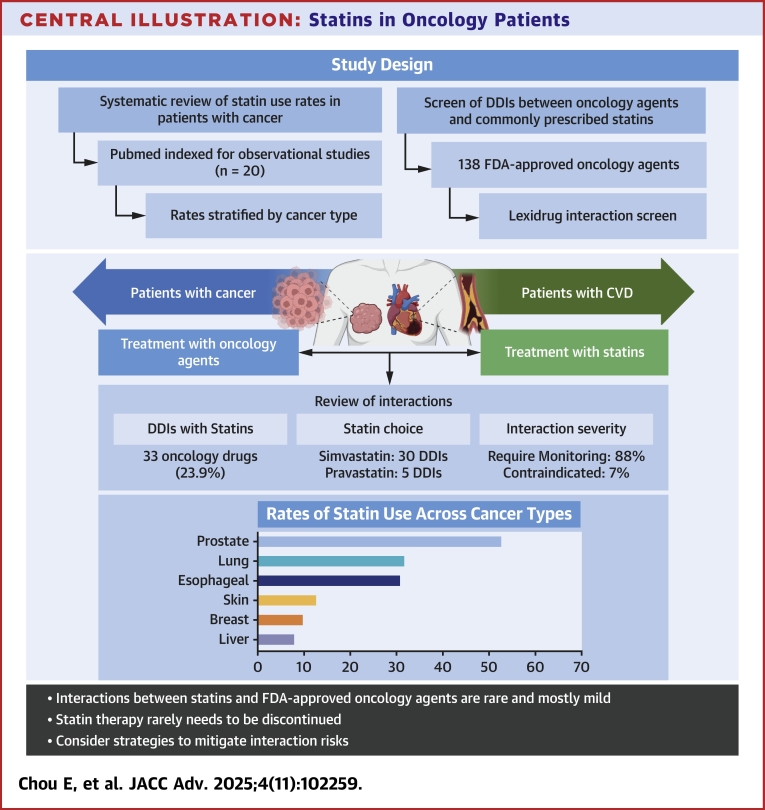
Statins in Oncology Patients Analysis of DDIs between statins and recently approved oncology agents, assessing severity and distribution across cancer types. Of 138 FDA-approved oncology agents, 33 (24%) exhibited DDIs with statins, with most interactions requiring monitoring. Simvastatin showed the highest interaction rate. Statin prescribing was highest in prostate and lung cancer, and lowest in liver and breast cancer. Findings suggest that DDIs between statins and oncology drugs are uncommon and rarely contraindicated, supporting the continuation of statins in cancer patients when clinically appropriate. CVD= cardiovascular disease; other abbreviations as in Figure 2.

## Funding support and author disclosures

Dr Baik has received consulting and advising fees from 10.13039/100016492Kiniksa Pharmaceuticals. All other authors have reported that they have no relationships relevant to the contents of this paper to disclose.
