# Supplementary material for: Statin Use in Patients With Cancer: Drug Interaction and Statin Usage
Source: JACC Adv. 2025 Oct 21;4(11):102259. doi: 10.1016/j.jacadv.2025.102259 (PMC12589975; doi:10.1016/j.jacadv.2025.102259)
Supplement: Supplemental Material [file mmc1.docx]

Supplemental Table 1: All interactions between FDA-approved oncology agents from the past 5 years and 5 most prescribed statins.

| Drug | Indications | Rosuva-statin | Atorva-statin | Simva-statin | Prava-statin | Lova-statin |
| --- | --- | --- | --- | --- | --- | --- |
| Abemaciclib | Breast |  |  |  |  |  |
| Abiraterone | Breast  Prostate | C | C | C | C | C |
| Acalabrutinib | CLL |  |  |  |  |  |
| Adagrasib | NSCLC  CRC |  | C | X |  | X |
| Alectinib | NSCLC |  |  |  |  |  |
| Amivantamab-vmjw | NSCLC |  |  |  |  |  |
| Apalutumide | Prostate | C | C | C |  | C |
| Asciminib | CML | X | X | C | C | C |
| Asparaginase erwinia chrysanthemi | Agnostic  Leukemia  Lymphoma |  |  |  |  |  |
| Atezolizumab | Breast  HCC  NSCLC  Melanoma  Sarcoma |  |  |  |  |  |
| Avapritinib | GIST  Mastocytosis |  |  |  |  |  |
| Avelumab | Urothelial |  |  |  |  |  |
| Axicabtagene ciloleucel | B cell lymphoma  Follicular lymphoma |  |  |  |  |  |
| Azacitidine | AML |  |  |  |  |  |
| Belantamab mafodotin-blmf | Multiple myeloma |  |  |  |  |  |
| Belzutifan | Agnostic  RCC |  |  |  |  |  |
| Bevacizumab | CRC  HCC  Ovarian, fallopian tube, primary peritoneal |  |  |  |  |  |
| Binimetinib | NSCLC |  |  |  |  |  |
| Blinatumomab | ALL |  |  |  |  |  |
| Brexucabtagene autoleucel | ALL  Mantle cell lymphoma |  |  |  |  |  |
| Brigatinib | NSCLC |  |  |  |  |  |
| Cabozantinib | RCC  Thyroid |  |  |  |  |  |
| Capivasertib | Breast |  |  | C |  |  |
| Capmatinib | NSCLC | D |  |  |  |  |
| Carboplatin | NSCLC |  |  |  |  |  |
| Carfilzomib | Multiple myeloma |  |  |  |  |  |
| Cedazuridine | Myelodysplastic syndroms |  |  |  |  |  |
| Cemiplimab-rwlc | NSCLC  Basal cell carcinoma |  |  |  |  |  |
| Cetuximab | CRC  Head/neck SCC^‡‡^ |  |  |  |  |  |
| Ciltacabtagene autoleucel | Multiple myeloma |  |  |  |  |  |
| Cisplatin | Urothelial carcinoma |  |  |  |  |  |
| Crizotinib | Anaplastic large cell lymphoma  Myelofibroblastic tumor |  | C | C |  | C |
| Dabrafenib | Agnostic |  | C | C |  | C |
| Daratumumab | Multiple myeloma |  |  |  |  |  |
| Darolutamide | Prostate | D | C | C | C | C |
| Decitabine | Myelodysplatic syndrome |  |  |  |  |  |
| Dostarlimab-gxly | Agnostic  Endometrial carcinoma |  |  |  |  |  |
| Durvalumab | Biliary tract  Endometrial carcinoma  Hepatocellular carcinoma  NSCLC  SCLC^§§^ |  |  |  |  |  |
| Eflornithine | Neuroblastoma |  |  |  |  |  |
| Elacestrant | Breast | C | C |  |  |  |
| Elranatamab-bcmm | Multiple myeloma |  |  | C |  |  |
| Encorafenib | NSCLC  CRC | C | C | C | C | C |
| Enfortumab vedotin-ejfv | Urothelial carcinoma |  |  |  |  |  |
| Entrectinib | Agnostic |  |  |  |  |  |
| Envatinib | Endometrial carcinoma |  |  |  |  |  |
| Enzalutamide | Prostate |  | C | C |  | C |
| Epcoritamab-bysp | B cell lymphoma  DLBCL^‖‖^  Follicular lymphoma |  |  | C |  |  |
| Erdafitinib | Urothelial carcinoma |  |  |  |  |  |
| Erlotinib | NSCLC |  |  | C |  |  |
| Fam-trastuzumab deruxtecan-nxki | Agnostic  Breast  NSCLC |  |  |  |  |  |
| Fedratinib | Myelofibrosis |  | C | C |  | C |
| Fruquintinib | CRC |  |  |  |  |  |
| Fulvestrant | Breast |  |  |  |  |  |
| Futibatinib | Cholangiocarcinoma | C | C |  |  |  |
| Gemcitabine | Urothelial carcinoma |  |  |  |  |  |
| Glofitamab-gxbm | B cell lymphoma |  |  | C |  |  |
| Hyaluronidase-zzxf | Breast |  |  |  |  |  |
| Ibrutinib | CLL |  |  |  |  |  |
| Idecabtagene vicleucel | Multiple myeloma |  |  |  |  |  |
| Imetelstat | Myelodysplastic syndrome |  |  |  |  |  |
| Infigratinib | Cholangiocarcinoma |  |  |  |  |  |
| Ipilimumab | Esophageal SCC  HCC  Mesothelioma  NSCLC |  |  |  |  |  |
| Irinotecan liposome | Pancreatic |  |  |  |  |  |
| Isatuximab-irfc | Multiple myeloma |  |  |  |  |  |
| Ivosidenib | Cholangiocarcinoma  AML  Myelodysplastic syndrome |  |  |  |  |  |
| Lenalidomide | Multiple myeloma |  |  |  |  |  |
| Lenvatinib | RCC |  |  |  |  |  |
| Lifileucel | Melanoma |  |  |  |  |  |
| Lisocabtagene maraleucel | B cell lymphoma  Follicular lymphoma  Mantel cell lymphoma |  |  |  |  |  |
| Loncastuximab tesirine-lpyl | B cell lymphoma |  |  |  |  |  |
| Lorlatinib | NSCLC |  | C | C |  | C |
| Lurbinectedin | SCLC |  |  |  |  |  |
| Luspatercept-aamt | Myelodysplastic syndrome |  |  |  |  |  |
| Margetuximab | Breast |  |  |  |  |  |
| Melphalan | Melanoma |  |  |  |  |  |
| Melphalan flufenamide | Multiple myeloma |  |  |  |  |  |
| Mirvetuxmab soravtansine-gynx | Ovarian  Fallopian tube  Primary peritoneal |  |  |  |  |  |
| Mitomycin | Urothelial carcinoma |  |  |  |  |  |
| Mobocertinib | NSCLC |  |  |  |  |  |
| Mosunetuzumab-axgb | Follicular lymphoma |  |  | C |  |  |
| Nadofaragene firadenovec-vncg | Bladder |  |  |  |  |  |
| Naxitamab | Neuroblatoma |  |  |  |  |  |
| Neratinib | Breast |  |  |  |  |  |
| Niraparib | Ovarian  Fallopian tube  Primary peritoneal  Prostate |  |  |  |  |  |
| Nirogacestat | Desmoid tumor |  | C | C |  | C |
| Nivolumab | Urothelial carcinoma  Esophageal or GEJ Adenocarcinoma  Esophageal SCC  HCC  Mesothelioma  NSCLC  Melanoma  RCC |  |  |  |  |  |
| Nogapendekin alfa inbakicept-pmln | Bladder |  |  |  |  |  |
| Olaparib | Breast  Ovarian  Fallopian tube  Primary peritoneal  Prostate  Pancreatic |  |  |  |  |  |
| Olutasidenib | AML |  |  |  |  |  |
| Omidubicel | Hematologic malignancies |  |  |  |  |  |
| Osimertinib | NSCLC | C | C | B |  |  |
| Paclitaxel | Breast  NSCLC |  |  |  |  |  |
| Pembrolizumab | Agnostic  Biliary  Bladder  Breast  Cervical  CRC  Endometrial carcinoma  Esophageal or GEJ Adenocarcinoma  Esophageal SCC  Gastric  NSCLC  Hodgkin lymphoma  Melanoma  RCC  SCC |  |  |  |  |  |
| Pemigatinib | Agnostic  Cholangiocarcinoma |  |  |  |  |  |
| Pertuzumab | Breast |  |  |  |  |  |
| Pexidartinib | Tenosyvial giant cell tumor |  | C | C |  | C |
| Pirtobrutinib | CLL  Mantle cell lymphoma | C |  | C |  |  |
| Pluvicto | Prostate |  |  |  |  |  |
| Polatuzumab vedotin-piiq | B cell lymphoma  DLBCL |  |  |  |  |  |
| Pomalidomide | Kaposi sarcoma  Multiple myeloma |  |  |  |  |  |
| Ponatinib | ALL |  |  |  |  |  |
| Pralsetinib | NSCLC  Thyroid |  |  |  |  |  |
| Quizartinib | AML |  |  |  |  |  |
| Relatlimab (opdualag) | Melanoma |  |  |  |  |  |
| Relugolix | Prostate |  |  |  |  |  |
| Repotrectinib | Agnostic  NSCLC |  | C | C |  | C |
| Retifanlimab-dlwr | Merkel cell carcinoma |  |  |  |  |  |
| Ripretinib | GIST |  |  |  |  |  |
| Rituximab | CLL |  |  |  |  |  |
| Rucaparib | Prostate | B |  | C |  |  |
| Sacituzumab govitecan-hziy | Urothelial carcinoma  Breast Cancer |  |  |  |  |  |
| Selinexor | Multiple myeloma |  |  |  |  |  |
| Selpercatinib | Thyroid  Agnostic  NSCLC |  |  | C |  |  |
| Sirolimus protein bound | Ovarian  Fallopian tube  Primary peritoneal |  |  |  |  |  |
| Sotorasib | NSCLC | C | C | C |  | C |
| Tafasitamab-cxix | DLBCL |  |  |  |  |  |
| Talazoparib | Prostate |  | C |  |  |  |
| Talquetamab-tgvs | Multiple myeloma |  |  | C |  |  |
| Tarlatamab-dlle | SCLC |  |  | C |  |  |
| Tazemetostat | Follicular lymphoma  Epithelioid sarcoma |  |  |  |  |  |
| Tebentafusp-tebn | Melanoma |  |  |  |  |  |
| Teclistamab-cqyv | Multiple myeloma |  |  | C |  |  |
| Tepotinib | NSCLC |  |  |  |  |  |
| Tipiracil | CRC |  |  |  |  |  |
| Tisagenlecleucel | Follicular lymphoma |  |  |  |  |  |
| Tisotumab vedotin-tftv | Cervical |  |  |  |  |  |
| Tivozanib | RCC |  |  |  |  |  |
| Toripalimab-tpzi | Nasopharyngeal carcinoma |  |  |  |  |  |
| Trametinib | Agnostic |  |  |  |  |  |
| Trastuzumab | Breast  CRC |  |  |  |  |  |
| Trastuzumab deruxtecan-nxki | Esophageal or GEJ Adenocarcinoma |  |  |  |  |  |
| Tremelimumab | HCC  NSCLC |  |  |  |  |  |
| Trifluridine | CRC |  |  |  |  |  |
| Tucatinib | Breast  CRC |  | C | X |  | X |
| Umbralisib | Marginal zone lymphoma  Follicular lymphoma |  |  |  |  |  |
| Venetoclax | AML |  |  |  |  |  |
| Zanubrutinib | CLL  Follicular lymphoma  Mantle cell lymphoma  Marginal lymphoma |  |  |  |  |  |

ALL = Acute lymphoid leukemia; AML = Acute myeloid leukemia; CLL = Chronic lymphoid leukemia; CML = Chronic myeloid leukemia; CRC = Colorectal cancer; DLBCL = Diffuse large B-cell lymphoma; GEJ = Gastroesophageal junction; GIST = Gastrointestinal stromal tumor; HCC = Hepatocellular carcinoma; NSCLC = Non-small cell lung cancer; RCC = Renal cell carcinoma; SCC = Squamous cell carcinoma; SCLC = Small cell lung cancer

Supplemental Table 2: Additional study characteristics.

| Study | Newcastle Ottawa scale | Age reporting method | Statin users | Statin non-users |
| --- | --- | --- | --- | --- |
| Lotsch et al. 2014 | 9.00 | Median (IQR) | 65 (61-70) | 60 (51-67) |
| Chan et al. 2015 | 9.00 | Mean (SD) | 69.1 (7.4) | 70.7 (6.8) |
| Shao et al. 2015 | 7.00 | Mean | 59.41 | 63.76 |
| Chen et al. 2016 | 9.00 | Mean (SD) | 65.91 (10.25) | 65.80 (10.37) |
| Wang et al. 2016 | 8.00 | Age categories | <50-59: 32.8%  60-69: 44.97%  >70: 22.3% | <50-59: 16.4%  60-69: 52.62%  >70: 30.94% |
| Wu et al. 2016 | 9.00 | Mean | 64.3 | 65.21 |
| Mikkelsen et al. 2017 | 8.00 | Median | 74 | 75 |
| Palumbo et al. 2017 | 7.00 | Median (Range) | 74 (57-85) ^a^ | |
| Emilsson et al. 2018 | 9.00 | Mean (SD) | 76.4 (7.4) ^a^ | |
| Anderson Carter et al. 2019 | 8.00 | Median (IQR) | 73 (67-78) | 76 (70-81) |
| Fransgaard et al. 2019 | 8.00 | Age categories | <60: 17.6%  61-70: 44.6%  71-80: 35.2%  >80: 9% | <60: 18.8%  61-70: 42.9%  71-80: 36.7%  >80: 1.7% |
| Jimenez-Vacas et al. 2020 | 8.00 | Median (IQR) | 61 (56-66) ^a^ | |
| Majidi et al. 2020 | 9.00 | Mean (SD) | 66.9 (7.4) | 58.2 (10.9) |
| Fernandez et al. 2021 | 9.00 | Median (IQR) | 63 (58, 68) | 58 (50, 63) |
| Okada et al. 2021 | 9.00 | Mean (SD) | 64 (10) | 65 (9) |
| Rossi et al. 2021 | 9.00 | Mean (Range) | 71 (48-93) | 70 (46-90) |
| Chung et al. 2022 | 8.00 | Mean (SD) | 61.7 (11.45) ^b^ | |
| Pourlotfi et al. 2022 | 9.00 | Mean (SD) | 71.5 (12) | 74 (8) |
| Okamoto et al. 2023 | 7.00 | Median (Range) | 69 (43-79) | 56 (26-82) |
| Lin et al. 2024 | 9.00 | Mean (SD) | 56.35 (8.10) ^b^ | |

^a^ Age not reported separately for subgroup of statin use

^b^When age was reported separately for subgroups other than statin use, overall mean ± SD was calculated using pooled formulas

Supplemental Table 3: Rates of statin use in patients with cancer stratified by cancer stage

| Study | Cancer Type | Stage I | Stage II | Stage III | Stage IV |
| --- | --- | --- | --- | --- | --- |
| Mikkelsen et al. 2017 | Prostate | 19 / 31 (61.3%) | 53 / 169 (31.4%) | 55 / 145 (37.9%) | 8 / 30 (26.7%) |
| Pourlotfi et al. 2022 | Colorectal | 1293 / 3620 (35.7%) | 2577 / 8452 (30.5%) | 2026 / 7046 (28.7%) | N/A |
| Majidi et al. 2020 | Ovarian | 27 / 188 (14.4%) | 12 / 82 (14.6%) | 131 / 563 (23.3%) | 29 / 122 (23.8%) |
| Fransgaard et al. 2019 | Colorectal | 57 / 115 (49.6%) | 99 / 191 (51.8%) | 166 / 333 (49.8%) | 14 / 27 (51.9%) |

Supplemental Table 4: Statin use by type in patients with cancer

| Study | Atorvastatin | Simvastatin | Pravastatin | Lovastatin | Fluvastatin | Rosuvastatin | Pitavastatin |
| --- | --- | --- | --- | --- | --- | --- | --- |
| Lotsch et al. 2014 | 48 / 158 (30.4%) | 96 / 158 (60.8%) | 14 / 158 (8.9%) | 1 / 158 (0.6%) | 5 / 158 (3.2%) | 6 / 158 (3.8%) | 0 / 158 (0.0%) |
| Wang et al. 2016 | 514 / 1529 (33.6%) | 436 / 1529 (28.5%) | 241 / 1529 (15.8%) | 192 / 1529 (12.6%) | 113 / 1529 (7.4%) | 0 / 1529 (0.0%) | 0 / 1529 (0.0%) |
| Fernandez et al. 2021 | 50 / 113 (44.2%) | 36 / 113 (31.9%) | 12 / 113 (10.6%) | 2 / 113 (1.8%) | 0 / 113 (0.0%) | 11 / 113 (9.7%) | 2 / 113 (1.8%) |
| Okamoto et al. 2023 | 7 / 55 (12.7%) | 0 / 55 (0.0%) | 5 / 55 (9.1%) | 0 / 55 (0.0%) | 1 / 55 (1.8%) | 11 / 55 (20.0%) | 7 / 55 (12.7%) |
| **Total** | 619 / 1855 (33.4%) | 568 / 1855 (30.6%) | 272 / 1855 (14.7%) | 195 / 1855 (10.5%) | 119 / 1855 (6.4%) | 28 / 1855 (1.5%) | 9 / 1855 (0.5%) |

**Preferred Reporting Items for Systematic reviews and Meta-Analyses extension for Scoping Reviews (PRISMA-ScR) Checklist**

| **SECTION** | **ITEM** | **PRISMA-ScR CHECKLIST ITEM** | **REPORTED ON PAGE #** |
| --- | --- | --- | --- |
| **TITLE** | | | |
| Title | 1 | Identify the report as a scoping review. | 1 |
| **ABSTRACT** | | | |
| Structured summary | 2 | Provide a structured summary that includes (as applicable): background, objectives, eligibility criteria, sources of evidence, charting methods, results, and conclusions that relate to the review questions and objectives. | 2 |
| **INTRODUCTION** | | | |
| Rationale | 3 | Describe the rationale for the review in the context of what is already known. Explain why the review questions/objectives lend themselves to a scoping review approach. | 5 |
| Objectives | 4 | Provide an explicit statement of the questions and objectives being addressed with reference to their key elements (e.g., population or participants, concepts, and context) or other relevant key elements used to conceptualize the review questions and/or objectives. | 5 |
| **METHODS** | | | |
| Protocol and registration | 5 | Indicate whether a review protocol exists; state if and where it can be accessed (e.g., a Web address); and if available, provide registration information, including the registration number. | 8 |
| Eligibility criteria | 6 | Specify characteristics of the sources of evidence used as eligibility criteria (e.g., years considered, language, and publication status), and provide a rationale. | 7 |
| Information sources* | 7 | Describe all information sources in the search (e.g., databases with dates of coverage and contact with authors to identify additional sources), as well as the date the most recent search was executed. | 7 |
| Search | 8 | Present the full electronic search strategy for at least 1 database, including any limits used, such that it could be repeated. | 7 |
| Selection of sources of evidence† | 9 | State the process for selecting sources of evidence (i.e., screening and eligibility) included in the scoping review. | 7 |
| Data charting process‡ | 10 | Describe the methods of charting data from the included sources of evidence (e.g., calibrated forms or forms that have been tested by the team before their use, and whether data charting was done independently or in duplicate) and any processes for obtaining and confirming data from investigators. | 7 |
| Data items | 11 | List and define all variables for which data were sought and any assumptions and simplifications made. | 7 |
| Critical appraisal of individual sources of evidence§ | 12 | If done, provide a rationale for conducting a critical appraisal of included sources of evidence; describe the methods used and how this information was used in any data synthesis (if appropriate). | 8 |
| Synthesis of results | 13 | Describe the methods of handling and summarizing the data that were charted. | 7,8 |
| **RESULTS** | | | |
| Selection of sources of evidence | 14 | Give numbers of sources of evidence screened, assessed for eligibility, and included in the review, with reasons for exclusions at each stage, ideally using a flow diagram. | 10 |
| Characteristics of sources of evidence | 15 | For each source of evidence, present characteristics for which data were charted and provide the citations. | 10 |
| Critical appraisal within sources of evidence | 16 | If done, present data on critical appraisal of included sources of evidence (see item 12). | 8 |
| Results of individual sources of evidence | 17 | For each included source of evidence, present the relevant data that were charted that relate to the review questions and objectives. | 11 |
| Synthesis of results | 18 | Summarize and/or present the charting results as they relate to the review questions and objectives. | 10, 11 |
| **DISCUSSION** | | | |
| Summary of evidence | 19 | Summarize the main results (including an overview of concepts, themes, and types of evidence available), link to the review questions and objectives, and consider the relevance to key groups. | 14 |
| Limitations | 20 | Discuss the limitations of the scoping review process. | 15 |
| Conclusions | 21 | Provide a general interpretation of the results with respect to the review questions and objectives, as well as potential implications and/or next steps. | 14 |
| **FUNDING** | | | |
| Funding | 22 | Describe sources of funding for the included sources of evidence, as well as sources of funding for the scoping review. Describe the role of the funders of the scoping review. | 1 |

JBI = Joanna Briggs Institute; PRISMA-ScR = Preferred Reporting Items for Systematic reviews and Meta-Analyses extension for Scoping Reviews.

* Where *sources of evidence* (see second footnote) are compiled from, such as bibliographic databases, social media platforms, and Web sites.

† A more inclusive/heterogeneous term used to account for the different types of evidence or data sources (e.g., quantitative and/or qualitative research, expert opinion, and policy documents) that may be eligible in a scoping review as opposed to only studies. This is not to be confused with *information sources* (see first footnote).

‡ The frameworks by Arksey and O’Malley (6) and Levac and colleagues (7) and the JBI guidance (4, 5) refer to the process of data extraction in a scoping review as data charting*.*

§ The process of systematically examining research evidence to assess its validity, results, and relevance before using it to inform a decision. This term is used for items 12 and 19 instead of "risk of bias" (which is more applicable to systematic reviews of interventions) to include and acknowledge the various sources of evidence that may be used in a scoping review (e.g., quantitative and/or qualitative research, expert opinion, and policy document).

*From:* Tricco AC, Lillie E, Zarin W, O'Brien KK, Colquhoun H, Levac D, et al. PRISMA Extension for Scoping Reviews (PRISMAScR): Checklist and Explanation. Ann Intern Med. 2018;169:467–473. [doi: 10.7326/M18-0850](http://annals.org/aim/fullarticle/2700389/prisma-extension-scoping-reviews-prisma-scr-checklist-explanation).
